# MicroRNAs 99b-5p/100-5p Regulated by Endoplasmic Reticulum Stress are Involved in Abeta-Induced Pathologies

**DOI:** 10.3389/fnagi.2015.00210

**Published:** 2015-11-18

**Authors:** Xiaoyang Ye, Hongxue Luo, Yan Chen, Qi Wu, Yi Xiong, Jinyong Zhu, Yarui Diao, Zhenguo Wu, Jianting Miao, Jun Wan

**Affiliations:** ^1^Shenzhen Key Laboratory for Neuronal Structural Biology, Biomedical Research Institute, Shenzhen Peking University – The Hong Kong University of Science and Technology Medical Center, Shenzhen, China; ^2^Division of Life Science, The Hong Kong University of Science and Technology, Hong Kong, China; ^3^Ludwig Institute for Cancer Research, San Diego, CA, USA; ^4^Department of Neurology, Tangdu Hospital, Fourth Military Medical University, Xi’an City, China

**Keywords:** microRNA-99b-5p, microRNA-100-5p, amyloid β, Alzheimer’s disease, endoplasmic reticulum stress

## Abstract

Alzheimer’s disease (AD) is the most common cause of dementia. Amyloid β (Abeta, Aβ) deposition and intracellular tangles are the pathological hallmarks of AD. MicroRNAs (miRNAs) are small non-coding RNAs, which have been found to play very important roles, and have the potential to serve as diagnostic markers during neuronal pathogenesis. In this study, we aimed to determine the roles of miR-99b-5p and miR-100-5p in Aβ-induced neuronal pathologies. We detected the expression levels of miR-99b-5p and miR-100-5p in the brains of APPswe/PS1ΔE9 double-transgenic mice (APP/PS1 mice) at different age stages and found that both miRNAs were decreased at early stages while increased at late stages of APP/PS1 mice when compared with the age-matched wild type (WT) mice. Similar phenomenon was also observed in Aβ-treated cultured cells. We also confirmed that mammalian target of rapamycin (mTOR) is one of the targets of miR-99b-5p/100-5p, which is consistent with previous studies in cancer. MiR-99b-5p/100-5p has been found to promote cell apoptosis with the Aβ treatment. This effect may be induced via the mTOR pathway. In our study, we find both miR-99b-5p and miR-100-5p affect neuron survival by targeting mTOR. We also speculate that dynamic change of miR-99b-5p/100-5p levels during Aβ-associated pathologies might be attributed to Aβ-induced endoplasmic reticulum stress (ER stress), suggesting the potential role of the “ER stress–miRNAs–mTOR” axis in Aβ-related AD pathogenesis.

## Introduction

Alzheimer’s disease (AD) is the most common human neurodegenerative disease in the elderly; more than 30 million people suffer from AD worldwide (Schonrock et al., 2012). The nosogenesis of AD is widely accepted as amyloid-β peptide (Aβ) deposition caused by abnormal protein metabolism and tau protein precipitation in AD brains. The AD progress encompasses multiple alterations of gene expression and protein reactions related to Aβ deposition, tau hyperphosphorylation, inflammation, energy metabolism, cell cycle, and apoptosis (Ballard et al., 2009; Schonrock et al., 2012). Aβ is the main component of amyloid plaques, which trigger cascaded reaction, leading to synaptic dysfunction, disrupting neural connectivity and neuronal death (Murphy and LeVine, 2010). Recently, lots of studies have demonstrated that aberrant levels of microRNAs (miRNAs) are widely involved in AD brains, indicating a complicated regulatory network of miRNAs in the etiopathology of AD (Schratt et al., 2006; Cheng et al., 2009; Magill et al., 2010; Zhao et al., 2010).

MicroRNAs are non-coding RNAs with 21–25 nt, which originate from both intergenic and intronic regions, inhibiting protein expression in a post-transcriptional manner by interacting with the 3′-UTR of target mRNAs (Ambros, 2004). A single miRNA has the ability to target thousands of mRNA molecules. Moreover, there are hundreds of potential miRNA-binding sites in just one mRNA molecule. MiRNAs have been reported to be involved in a wide spectrum of human diseases, especially in cancer, infectious diseases, cardiac diseases, and many others (Ardekani and Naeini, 2010; Tufekci et al., 2014). Accumulating evidences show that miRNAs also participate in numerous neurodegenerative disorders, including AD, Amyotrophic lateral sclerosis, Parkinson’s disease, and Huntington’s disease (Kim et al., 2007). Recent studies of AD have demonstrated aberrant miRNA expression profile in AD via developed sequencing approach (Nunez-Iglesias et al., 2010; Shioya et al., 2010). The roles of miRNAs in APP and Aβ production, synaptic remodeling, neuron survival, and glia cell activation have also been identified (Kurosinski and Gotz, 2002; Kocerha et al., 2009; Schratt, 2009). Nevertheless, there are far more miRNAs remaining enigmatic as to their roles in AD etiology.

Our previous study found that miR-99b-5p and miR-100-5p were abnormally expressed in the brains of APPswe/PS1ΔE9 double-transgenic mice [APP/PS1 mice (Luo et al., 2014)], which have AD symptoms after 6 months of age, suggesting their pivotal roles in Aβ deposition-associated AD pathology. MiR-99b-5p and miR-100-5p belong to the same miR-99 family, which consists of three members, miR-99a, miR-99b, and miR-100. Studies have shown that miR-99 family regulates cell survival, cell stress response, proliferation, angiogenesis, DNA damage, and wound healing process (Zhang et al., 2009; Doghman et al., 2010; Sun et al., 2011; Chen et al., 2012; Zheng et al., 2012; Jin et al., 2013; Mueller et al., 2013). It has been reported that mammalian target of rapamycin (mTOR) is one of the targets of both miR-99 and miR-100 (Sun et al., 2011; Li et al., 2013; Xu et al., 2013).

Mammalian target of rapamycin is a conserved serine– threonine kinase, belonging to the phosphatidylinositol 3-kinase (PI3K)-related kinase protein family. This kinase family plays essential roles in cell apoptosis, proliferation and differentiation, cell senescence, cytoskeleton composition, angiogenesis, gene transcription, tumor formation, and development, mainly via PI3K/Akt/mTOR signaling pathway (Hassan et al., 2013; Maiese et al., 2013; Trigka et al., 2013). Interestingly, emerging evidences demonstrate that mTOR regulates neuron survival, neuronal protection, autophagy, synaptic development, and function in nerve system (Chen et al., 2010; Heras-Sandoval et al., 2014). Previous studies have illustrated that both in patient cases and mouse models of AD, Aβ can inhibit mTOR pathway, leading to dysregulation of tau, phosphatase and tensin homolog (PTEN), and neuronal survival as well as plasticity (Chen et al., 2010). In addition, mTOR is associated with clearance of Aβ, synaptic remodeling, long-term potentiation (LTP), and cognitive decline (Jaworski et al., 2005; Paccalin et al., 2006; Hoeffer and Klann, 2010; Ma et al., 2010), indicating its predominant roles in AD development. Therefore, the role of mTOR under the regulation of miR-99b-5p and miR-100-5p in Aβ-induced neuronal pathologies needs to be further illuminated.

In this study, we used APP/PS1 mice to determine the role of miR-99b-5p and miR-100-5p in Aβ-associated brain pathologies. Our work first demonstrated that miR-99b-5p/100-5p levels exhibited a dynamic change along the Aβ deposition, which could also be recapitulated in Aβ-treated cells *in vitro*. We presumed that this dynamic change of both miRNAs might be due to Aβ-induced endoplasmic reticulum stress (ER stress). Moreover, we found that miR-99b-5p/100-5p played a pivotal role via targeting mTOR, indicating that the ER stress–miRNAs–mTOR axis might give us a novel clue to understand Aβ-induced AD pathology.

## Materials and Methods

### Animal Samples

The APP/PS1 mice [held by The Jackson Laboratory, stain name: “B6.Cg-Tg(APPswe, PSEN1dE9)85Dbo/Mmjax” (Borchelt et al., 1996)] were purchased from the Model Animal Research Center of Nanjing University. The non-carrier C57BL/6J mice were used as the wild type (WT) control. Six to eight pairs of APP/PS1 and WT mice at different age stages were included in this study. All the mice were anesthetized with pentobarbital (50 mg/kg). Then, 0.5 ml of peripheral whole blood was kept in anticoagulation tubes with EDTA. Cortexes of the brains were dissected and lysed for either RNA or protein extraction. All the animal experiments were performed in accordance with animal use guidelines approved by the Committee for the Ethics of Animal Experiment, Shenzhen-Peking University, The Hong Kong University of Science and Technology Medical Center (SPHMC). The protocol number is 2011-004.

### Reagents and Antibodies

Amyloid-β peptide 1–42 (Aβ1–42) was from AnaSpec (AS-20276). The ER stress inhibitor sodium phenylbutyrate (PBA) and inducer thapsigargin (TG) were both from Sigma. PBA and TG were dissolved in ethanol and used with the final concentration of 10 mM and 500 nM, respectively. MiRNA mimics and inhibitors were purchased from Invitrogen. Mouse monoclonal antibodies of GAPDH and β-actin were from Sangon, and rabbit polyclonal antibody of mTOR was from Cell Signaling Technology.

### Cell Culture and Aβ Treatment

Rat pheochromocytoma cells – PC12 (ATCC) were routinely cultured in DMEM (GIBCO) containing 6% fetal bovine serum (Hyclone), 6% horse serum (GIBCO), 50 U/ml penicillin, and 50 μg/ml streptomycin (GIBCO) in a humidified incubator at 37°C with 7.5% CO_2_. PC12 cell differentiation was induced by NGF. Cells were plated 1 day before treatment with NGF. After incubating for 1 day, culture medium was changed to differentiation medium (DMEM with 50 ng/ml NGF, 0.5% fetal bovine serum, 0.5% horse serum and penicillin/streptomycin). Primary cortical neuron cultures were prepared from embryonic day 18 rat embryos (Wan et al., 2008). Cortical neurons were plated and cultured on poly-L-lysine (PLL)-precoated culture plates in Neurobasal A medium containing B27 supplement, 0.05 mM glucose, and 0.5 mM L-glutamine in a 37°C, 5% CO_2_ incubator. Culture medium was half-changed every 2 days. Aβ1–42 oligomers were prepared by dissolving Aβ1–42 powder in 1% NH_4_OH followed by diluting to 0.2 mM in PBS, and incubating in 37°C water bath overnight. Aβ1–42 with different final concentrations was treated with cortical neurons cultured at 7-day *in vitro* (DIV7) or differentiated PC12 cells (NGF treatment for 48 h) for various time intervals as indicated.

### Plasma MicroRNAs Quantification Based on Taqman Probe qPCR

Whole blood samples from different ages of mice were centrifuged at 2,000 rpm for 10 min at room temperature. The separated plasma was stored at −80°C. RNA was isolated from 150 μl plasma by Trizol Reagent (Invitrogen) for Taqman probe-based (Invitrogen) qPCR analysis as described before (Wang et al., 2015). Both miR-99b-5p and miR-100-5p mimics were used to establish standard curves for the determination of the miRNAs copies in plasma. All probe-based qPCR was done using Lightcycler 480 probe Master (Roche) according to the manufacturer’s instructions. For normalization of sample variation, 5 ng internal control Mmu-miR-486 and external miRNAs control Cel-miR-238 were added to each plasma sample for extraction and qRT-PCR. The statistical analyses were performed using the methods as described before (Wang et al., 2015). The probe sequences were listed in Table 1.

**Table 1:** **Sequence of the primers used for real-time PCR**.

Primer/probe	Sequence
Has miR-R	5′-GTGCGTGTCGTGGAGTC-3′
snRNAU6-F	5′-GCTTCGGCAGCACATATACTAAAAT-3′
snRNAU6-R	5′-CGCTTCACGAATTTGCGTGTCAT-3′
miR-99b-5p-F	5′-CACCCGTAGAACCGACCTT-3′
miR-99b-5p-R	5′-GTCGTATCCAGTGCGTGTCGTGGAGTCGGCAATTGC ACTGGATACGACCGCAAGG-3′
miR-100-5p-F	5′-AAGAGAACCCGTAGATCCG-3′
miR-100-5p-R	5′-GTCGTATCCAGTGCGTGTCGTGGAGTCGGCAATTGC ACTGGATACGACCACAAG-3′
MsGAPDH-F	5′-AACTTTGGCATTGTGGAAGG-3′
MsGAPDH-R	5′-AACTTTGGCATTGTGGAAGG-3′
RatGAPDH-F	5′-TGTGAACGGATTTGGCCGTA-3′
RatGAPDH-R	5′-TGTGAACGGATTTGGCCGTA-3′
mTOR-F	5′-CTTCTTCCGTTCTATCTCCTT-3′
mTOR-R	5′-CTTCTTCCGTTCTATCTCCTT-3′
Mmu-miR-486-F	5′-ACCGTCCTGTACTGAGCT-3′
Mmu-miR-486-R	5′-GTCGTATCCAGTGCGTGTCGTGGAGTCGGCAATTGC ACTGGATACGACCTCGGG-3′
Cel-miR-238-F	5′-AGCCTTTGTACTCCGATGC-3′
Cel-miR-238-R	5′-GTCGTATCCAGTGCGTGTCGTGGAGTCGGCAATTGC ACTGGATACGACTCTGAA-3′
miR-99b-5p probe	5′-CACTGGATACGACCGCAAGGTCG-3′
miR-100-5p probe	5′-CACTGGATACGACCACAAGTTCGGT-3′

### RNA Extraction and Real-Time PCR

Total RNA was extracted by Trizol Reagent (Invitrogen) from either mice brains or cultured cells, followed by reverse transcription as described previously (Luo et al., 2014). CDNAs were then used as the templates in Real-time PCR using iQ™ SYBR^®^ Green Supermix (BIO-RAD). The mRNA level of mTOR was normalized by the average levels of GAPDH, while miRNA was by the levels of snRNAU6. The delta–deltaCt method was used in this study to quantify the fold change of both mRNA and miRNAs. The primer sequences were listed in Table 1.

### Transient Transfection

PC12 cells were plated on PLL-coated culture plates in growth medium at 16 h before the transfection. One hundred nanomole miRNAs (Invitrogen) were transfected into PC12 cells using Lipofectamine 2000 Reagent (Invitrogen) according to the manufacturer’s instruction. Immediately after isolated from the embryo cortexes, primary cortical neurons were transfected with the miRNAs using Neon™ MPK 5000S Transfection system (Invitrogen) according to the manufacturer’s protocol. The transfected neurons were plated on PLL-coated plates and cultured for 7 days before different treatments.

### Western Blot Assay

Brain tissues or cultured cells were lysed in RIPA buffer [150 mM NaCl, 1% (v/v) Nonidet P-40, 0.5% deoxycholic acid, 0.1% (w/v) SDS] containing protease inhibitors (cocktail, Sangon) and 1 mM phenylmethanesulfonyl fluoride (Sigma). The lysate was centrifuged at 4°C, 14,000 rpm for 15 min to get the supernatants. The proteins separated by SDS-PAGE were transferred onto PVDF membranes. GAPDH, β-actin, and mTOR antibodies were used in this study.

### Cell Viability Assays

To evaluated the cell viability, CellTiter 96 AQueous (Promega) was added to PC12 cells or primary cortical neurons at 1/10 volume of the medium. Cells were incubated at 37°C for 4 h. Two hundred microliters of mixed medium was transferred into 96-well ELISA plate. The absorbance was measured at 490 nm using a Microplate reader (BIO-RAD). Cell viability was presented as the percentage of absorbance obtained in control cells.

### Statistical Analysis

Data are represented as mean ± SD. Statistical analysis of the data was performed with Student’s *t* test using SPSS 13.0 software. A value of *p* < 0.05(*) was considered statistically significant.

## Results

### miR-99b-5p and miR-100-5p Are Involved in Aβ-Induced Alzheimer’s Disease

**Figure 1:**
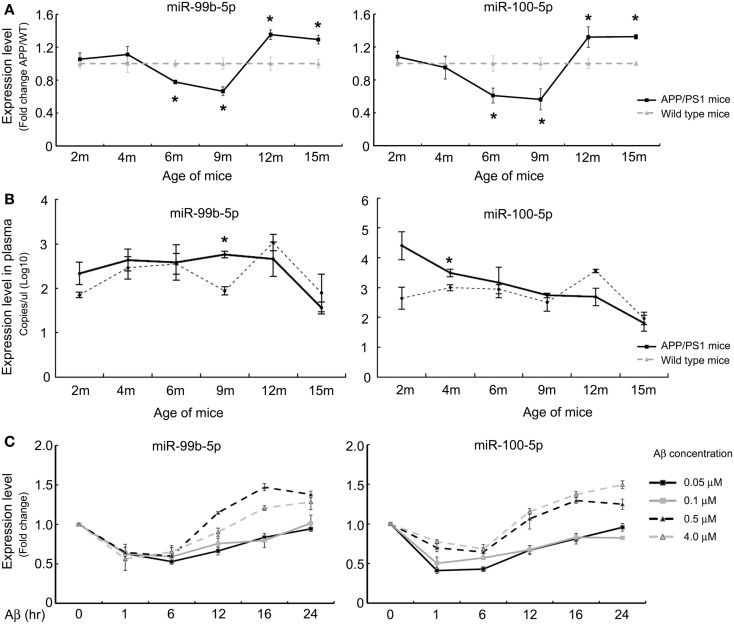
**The expression levels of miR-99b-5p and miR-100-5p in APP/PS1 mice and Aβ-treated PC12 cells**. **(A)** Relative expression levels (presented as fold change) of miR-99b-5p/miR-100-5p were quantified in six to eight pairs of APP/PS1 and WT mice cortexes by real-time PCR. The levels were normalized to the expression of snRNAU6 using the delta–deltaCt methods. **(B)** The absolute copy number of plasma miRNAs were calculated by referring to the standard curve and normalized with the Ct of mmu-miR-486 and cel-miR-238. Data are represented as mean ± SD, results were analyzed with Student’s *t* test (*p < 0.05, *n* = 3). **(C)** PC12 cells were differentiated for 48 h before the treatments with different concentrations of Aβ1–42 oligomers (0.05, 0.1, 0.5, and 4.0 μM) for various time intervals. Relative expression levels of miR-99b-5p and miR-100-5p are represented as fold change compared to vehicle controls. Data are expressed as mean ± SD and results were analyzed with Student’s *t* test [*p < 0.05, compared with control (*n* = 6)].

Our previous study demonstrated that miR-99b-5p/miR-100-5p was upregulated in 9-month-old APP/PS1 mice cortexes via high-throughput sequencing (Luo et al., 2014). To further explore the association of miR-99b-5p/miR-100 with Aβ-associated pathologies, real-time PCR was performed to determine the expression pattern along the aging progress in six to eight pairs of APP/PS1 and WT mice at each indicated age stage (2-, 4-, 6-, 9-, 12-, and 15-month-old). The expression levels of miR-99b-5p and miR-100-5p were significantly decreased at early stages (6 and 9 months) but increased at late stages (12 and 15 months) of APP/PS1 mice when compared with age-matched WT mice (Figure 1A), indicating that miR-99b-5p and miR-100-5p are dynamically regulated and exert their functions in accordance with different stages of brain pathologies induced by Aβ deposition.

In order to verify whether miR-99b-5p and miR-100-5p were detectable in mice plasma and thus could be used as biomarkers for diagnosis of AD, we detected the plasmatic copy numbers of two miRNAs via Taqman probe-based qPCR. After the normalization and calculation by referring to standard curve, we found that APP/PS1 mice showed higher expression levels of miR-99b-5p and miR-100-5p at 2, 4, 6, and 9 months in plasma, but lower at 12 months as well as 15 months (Figure 1B). This trend is opposite to the results in cortex tissues, indicating that the biogenesis, circulation, and distribution of miR-99b-5p and miR-100-5p along the development of Aβ-induced pathologies might be regulated by complicated yet sophisticated machinery.

To further investigate the expression pattern of two miRNAs after Aβ-treatment, we also used PC12 cell model. At the early stage of Aβ-treatment (6 h), miR-99b-5p/miR-100-5p levels were declined. Afterwards, the expression of both miRNAs was elevated, especially in the high concentration groups (Figure 1C), which is consistent with the data from mice tissues.

### mTOR Is One of the Targets of miR-99b-5p and miR-100-5p in PC12 Cells

**Figure 2:**
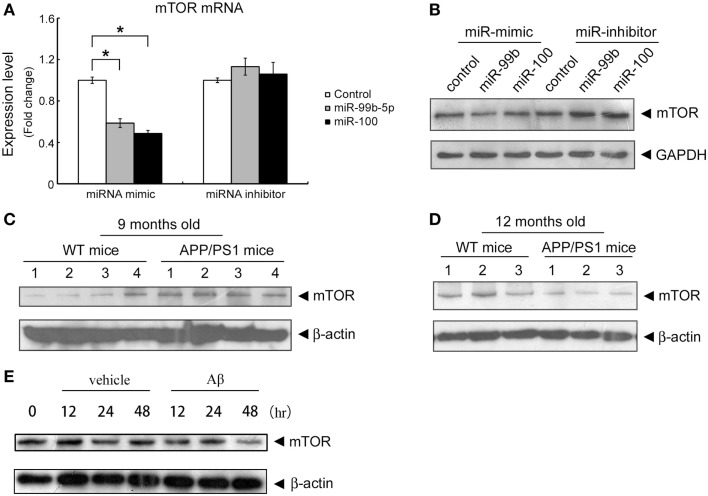
**Both miR-99b-5p and miR-100-5p negatively regulate mTOR expression in PC12 cells as well as in APP/PS1 mice**. **(A,B)** MiR-99b-5p and miR-100-5p mimics or inhibitors were transfected in PC12 cells. mTOR mRNA or protein levels were analyzed by real-time PCR or western blot, respectively. Data are represented as mean ± SD and results were analyzed with Student’s *t* test (*p < 0.05, *n* = 4). **(C,D)** Western blot analysis of mTOR protein level in cortex tissues from 9 and 12 months old APP/PS1 and WT mice. The housekeeping gene β-actin was taken as the internal reference. **(E)** Western blot of mTOR expression in PC12 at 0, 12, 24, and 48 h after the treatment of Aβ1-42.

In order to shed light on the functionality of miR-99b-5p and miR-100-5p in Aβ-induced pathologies, we predicted the potential targets of these two miRNAs using three online target prediction databases (miRDB, miRanda, and TargetScan) (Luo et al., 2014). Among the potential targets, mTOR was taken into consideration, which had been proved as one of the major targets of miR-99 family. Overexpression of miR-99b-5p and miR-100-5p dramatically reduced the mRNA and protein levels of mTOR in PC12 cells. In contrast, the miRNAs inhibitors only led to the upregulation effect on mTOR protein level but not mRNA level (Figures 2A,B). The expression level of mTOR protein in mice cortexes was also investigated. In 9-month-old APP/PS1 cortexes, mTOR level was higher than that in the age-matched WT mice cortex tissues, whereas it was lower in 12-month-old APP/PS1 cortexes than in the age-matched WT mice cortexes (Figures 2C,D). In Aβ-treated PC12 cells, the mTOR expression was also downregulated (Figure 2E). The inverse-correlated expression profile of miR-99b-5p/miR-100-5p and mTOR also indicates that miRNAs-regulated mTOR pathway may play different roles during the progression of brain injuries induced by Aβ deposition.

### miR-99b-5p and miR-100 Inhibit Cell Viability of Primary Neurons and PC12 Cells

**Figure 3:**
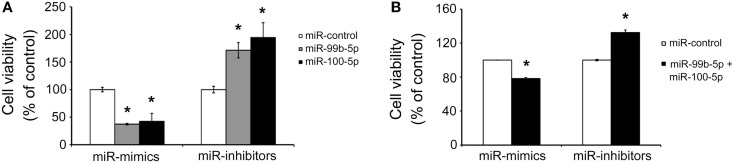
**MiR-99b-5p and miR-100-5p reduce cell viability**. **(A)** Cell viability assay in primary neurons with the transfection of miR-99b-5p/miR-100-5p mimics or inhibitors. **(B)** Co-transfection of miR-99b-5p and miR-100-5p mimics or inhibitors varied the cell viability in PC12 cells. Data are represented as mean ± SD; *n* = 3. Results were analyzed with Student’s *t* test (*p < 0.05).

To determine the role of miR-99b-5p and miR-100-5p in cell viability, we transfected the mimics or inhibitors of miR-99b-5p and miR-100-5p to primary rat cortical neurons or PC12 cells, respectively. Then the cells were treated with Aβ1–42 for 24 h. The viability of primary rat neurons was dramatically decreased when miR-99b-5p or miR-100-5p was overexpressed. On the contrary, cell viability was increased when miR-99b-5p or miR-100-5p was inhibited (Figure 3A). In PC12 cells, we also observed the similar results (Figure 3B), suggesting that either miR-99b-5p or miR-100-5p can further promote the neuronal cell death induced by Aβ1–42.

### The Expression Change of miR-99b-5p and miR-100-5p Induced by Aβ is Related to ER Stress

**Figure 4:**
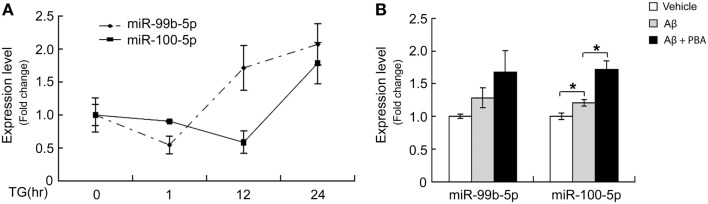
**ER stress regulates miR-99b-5p and miR-100-5p**. **(A)** NGF-differentiated PC12 cells were treated with TG (500 nM) for different time intervals as indicated. Total RNA was extracted. MiR-99b-5p and miR-100-5p were half-quantified by real-time PCR. **(B)** Aβ was treated to differentiated PC12 cells with or without PBA for 24 h, and the qRT-PCR was performed as **(A)**. Data are expressed as mean ± SD and results were analyzed with Student’s *t* test (*p < 0.05).

To determine whether the dynamic change of miR-99b-5p and miR-100-5p is related to Aβ-induced ER stress, we induced ER stress by TG in differentiated PC12 cells. Similar to Aβ treatment, TG also induced the decrease of two miRNAs at early stage but increase at late stage (Figure 4A). When we used ER stress inhibitor PBA to treat PC12 cells together with Aβ, the levels of miR-99b-5p and miR-100-5p after Aβ treatment were further upregulated (Figure 4B).

## Discussion

Many studies have showed the importance of mTOR signaling in brain. Neurons utilize mTOR to modulate multiple brain functions, including regulation of feeding, synaptic plasticity, and memory formation (Garelick and Kennedy, 2011). The cross talk of mTOR pathway with other signaling pathways has been indicated in autophagy, Aβ clearance, and AD development (Godoy et al., 2014). It has been demonstrated that the miR-99 can repress three targets: mTOR, SMARCA5 (SWI/SNF-related, matrix-associated, actin-dependent regulator of chromatin, subfamily a, member 5), and SMARCD1 (SWI/SNF-related, matrix-associated, actin-dependent regulator of chromatin, subfamily d, member 1) (Sun et al., 2011). mTOR, PLK1 (Polo-like kinase 1), FKBP51 (FK506 binding protein 51), IGF1R (insulin-like growth factor 1 receptor), IGF2 (insulin-like growth factor 2), and HOXA1 (homeobox A1) have been proved as the target genes of miR-100 (Nagaraja et al., 2010; Li et al., 2011, 2013; Gebeshuber and Martinez, 2013; Xu et al., 2013; Xiao et al., 2014). Here, mTOR is a common target of miR-99 and miR-100, and it is also a pivotal molecule involved in neurodegenerative diseases. To date, the functional role of mTOR in AD development is still enigmatic. However, several studies have suggested that inhibition of mTOR by rapamycin improves cognitive deficits and rescues Aβ pathology and NFTs through increased autophagy (Caccamo et al., 2010; Santos et al., 2011; Cai et al., 2012). In our study, we found that mTOR level was decreased at late stages of Aβ deposition mouse model cortexes. It is worthwhile to scrutinize whether mTOR plays positive roles in preventing neuronal death as early onset of Aβ deposition, and downregulation of mTOR at late stage ultimately leads to apoptosis of neuronal cells.

MicroRNA-99 family was found to be deregulated in different cancer types (Zhang et al., 2009; Doghman et al., 2010; Sun et al., 2011; Chen et al., 2012; Zheng et al., 2012; Jin et al., 2013; Mueller et al., 2013), but the role of miR-99 family in neuronal cell differentiation or cell death is not clear. Recently, Denk et al. (2015) found that miR-100 in cerebrospinal fluid (CSF) from AD patients could serve as one of the reliable biomarkers to detect AD. In this study, we chose APP/PS1 mice to explore the link between miR-99b-5p/miR-100-5p and Aβ-induced pathological development. Interestingly, the expression levels of miR-99b-5p and miR-100 in APP/PS1 mice brains were decreased at early stages (6–9 months old) and increased at late stages (12–15 months old) when compared with age-matched WT mice. This result suggests that miR-99b-5p/miR-100-5p play distinct roles during different stages of Aβ deposition-induced brain pathologies. To explain the underlying mechanism that orchestrates dynamic change of two miRNAs during the progression of Aβ-associated pathologies, we speculated ER stress as the regulator of the two miRNAs.

ER stress is triggered by the loss of homeostasis in the ER, leading to the accumulation of misfolded proteins within the ER lumen. Aβ was found to induce mild ER stress (Chafekar et al., 2007). We hypothesized that the change of miR-99b-5p/100-5p induced by Aβ was through ER stress induction, and we confirmed it by both activator and inhibitor of ER stress. The unfolded protein response (UPR) is a highly conserved stress response, functioning as a short-term adaptive mechanism to reduce unfolded protein levels and restore balance to the ER. However, if the UPR is insufficient to clear the unfolded proteins, another pathway, CHOP pathway, is activated, and ER stress-induced cell death occurs (Logue et al., 2013). APP and its processing products were found to have potential role in UPR (Endres and Reinhardt, 2013). Aβ can induce ER stress in both cultured neurons and animal models (Lee et al., 2010; Costa et al., 2013; Kondo et al., 2013). In our APP/PS1 mice, we could detect ER stress markers (PERK, eIF2α, and CHOP) at different stages, which were correlated with the change of miRNAs (data not shown). At UPR stages (6–9 months), the levels of miR-99b-5p/miR-100-5p were decreased, which were beneficial for neuron survival and synaptic plasticity. However, at late stages, due to long exposure to Aβ, the ER stress-induced death signaling was activated, and two miRNAs were increased to induce neuronal apoptosis. Three signaling pathways, IRE1α, PERK, and ATF6 pathways, modulate UPR upon ER stress induction. The downstream signals of all three pathways regulate the transcription of their target genes. We hypothesized that miR-99b-5p/miR-100-5p might also be one of the target genes. However, we still need more studies to verify our hypothesis.

Taken together, increasing evidences suggest that mTOR pathway may be a critical regulator of Aβ-associated pathologies. Our findings about the dynamic alteration of miR-99b-5p/100-5p-mTOR pathway during the progression of Aβ injuries provide further insight toward our understanding of Aβ-related AD pathogenesis. Recently, many scientists in the field are taking effort to look for new biomarkers for AD diagnosis, and circulating miRNAs have been regarded as promising and feasible approach in clinical. In our study, we also investigated the expression levels of miR-99b-5p/100-5p in the plasma of APP/PS1 mice. The differences of their levels between APP/PS1 and WT mice were not significant at some stages. It might be due to the small sample size included in our study. However, we still saw an interesting trend that plasma miRNA levels were inversely correlated with that of cortexes. Further investigations on animal models as well as AD patients are needed to address the underlying mechanism of how the pathological progression of AD in central nervous system affects the copy numbers of miRNAs in peripheral plasma in order to provide novel insights into non-invasive diagnostic methodology for AD.

## Author Contributions

XY, HL, ZW, JM, and JW conceived and designed the experiments. XY, HL, YC, QW, YX, JZ, and YD performed the experiments. XY, HL, YC, ZW, JM, and JW analyzed the data. XY, YC, JM, and JW wrote the paper.

## Conflict of Interest Statement

The authors declare that the research was conducted in the absence of any commercial or financial relationships that could be construed as a potential conflict of interest.

## Abbreviations

Aβ, amyloid β; AD, Alzheimer’s disease; APP, amyloid precursor protein; ER stress, endoplasmic reticulum stress; MiRNA, microRNA; mTOR, mammalian target of rapamycin; PI3K, phosphatidylinositol 3-kinase; PS1, presenilin 1; WT, wild type.
